# Upgrade procedures of pacemakers or implantable cardioverter-defibrillators require creative clinical decision making and careful planning that begins at the first implantation

**DOI:** 10.1093/europace/euag174

**Published:** 2026-09-25

**Authors:** Alexander H Maass, Hessel F Groenveld, Michiel Rienstra

**Affiliations:** Department of Cardiology, Heart Center, University of Groningen, University Medical Center Groningen, P.O. Box 30.001, Groningen 9700 RB, The Netherlands; Department of Cardiology, Heart Center, University of Groningen, University Medical Center Groningen, P.O. Box 30.001, Groningen 9700 RB, The Netherlands; Department of Cardiology, Heart Center, University of Groningen, University Medical Center Groningen, P.O. Box 30.001, Groningen 9700 RB, The Netherlands

To the editor:

Keene *et al*.^1^ presented their comprehensive clinical consensus statement from several professional rhythm societies on pacemaker or implantable cardioverter-defibrillator (ICD) upgrades and downgrades. We would like to congratulate the authors with their insightful review of this important topic.

While this manuscript covers all aspects of the subject matter, we would like to bring several points the attention of the authors and readers:

The consensus statement provides advice on how to act when an additional lead is needed because of additional pacing capabilities such as upgrade to cardiac resynchronization therapy (CRT).^2^ In our view the whole concept can be translated into strategies when an active pacemaker or ICD lead has become dysfunctional. Even though adding a new lead and deactivating a defective old lead is not technically an upgrade procedure, the same clinical decision making can be applied. The only exception might be that extracting a non-functional lead may be more readily considered to create access or reduce the risk for lead-lead interactions.

The first section of the workflow is about optimizing initial choice of the device. We would like to get the view of the authors about optimal choice of the electrode. In our view, there may still be a role for using a DF-1 lead. As downgrade procedures are limited by current hardware, we and others^3^ propose to consider a DF-1 lead in all patients scheduled for ICD with a pacing indication, including CRT patients. When upgrading from a bradycardia pacemaker to an ICD, it could be considered to use a DF-1 lead and connect the ventricular pacing electrode to the ICD when it shows acceptable electrical values. Using a functional pacemaker electrode has the advantage of reducing immediate postoperative risks such as lead dislocation or increasing thresholds. In addition, longevity of pacemaker leads might be much longer than that of ICD leads even if it has been active for many years.

In patients with a DF-4 lead that could be downgraded to a pacemaker, the additional risk of placing a new pacing lead weigh higher than the cost and size of an ICD and may be reserved for patients where the ventricular lead shows signs of electrical deterioration.

The last remarks concern patients with venous obstructions and we would like to get the view of the authors on the preferred strategy to overcome this problem. Venoplasty is increasingly used with high success rates. Our strategy has been published, and we would like to emphasize that venous access can be created from a peripheral (brachial) vein.^4^ This approach has the advantage of more working room for occlusions of the subclavian vein. In addition, the device pocket can remain closed until access has been established reducing infection risk but also obviating the need to escalate to lead extraction in the same session. The contralateral approach should be avoided as much as possible. Even though a recent manuscript has demonstrated safety of these procedures,^5^ in our experience virtually all patients with symptoms arising from vena cava superior syndrome that is not cause by cancer had bilateral pacemaker leads and/or other indwelling catheters.

We hope that our remarks can provide valuable additions to this comprehensive clinical consensus statement.

A.H.M. is chair of the Dutch Heart Registration (NHR) pacemaker and ICD committee and reports research funding for the DUTCH-ICD study by ZE&GG and ZonMW, grant number 10330052310001. All unrelated to the current manuscript.

## References

[1] Keene D, Nielsen JC, Burri H, Chavez-Gutierrez CA, Deharo JC, Drossart I et al Cardiac implantable electronic device upgrades and downgrades: a clinical consensus statement of the European Heart Rhythm Association (EHRA) of the ESC, the Asia Pacific Heart Rhythm Association (APHRS), Canadian Heart Rhythm Society (CHRS), Heart Rhythm Society (HRS), and the Latin American Heart Rhythm Society (LAHRS). Europace 2025;27:euaf252. 10.1093/europace/euaf252 PMC12696876 41378985

[2] Merkely B, Hatala R, Merkel E, Szigeti M, Veres B, Fabian A et al Benefits of upgrading right ventricular to biventricular pacing in heart failure patients with atrial fibrillation. Europace 2024;26:euae179. 10.1093/europace/euae179 PMC11264293 38979560

[3] Sticherling C, Ellenbogen KA, Burri H. Stepping back for good reasons: a reappraisal of the DF-1 connector for defibrillator leads. Europace 2024;26:euae057. 10.1093/europace/euae057 PMC10919383 38412340

[4] Lipsic E, Daniels F, Groenveld HF, Rienstra M, Maass AH. When and how to perform venoplasty for lead placement. Heart Rhythm 2024;21:1923–8. 10.1016/j.hrthm.2024.04.088 38692339

[5] Boyle TA, Callans D, Deo R, Garcia F, Guandalini G, Hyman MC et al Lead implantation in the setting of venous occlusion: long-term clinical outcomes with tunneled leads. JACC Clin Electrophysiol 2025;11:1616–8. 10.1016/j.jacep.2025.04.022 40536448

